# Mathematic Law of Digestive Activity

**Published:** 1915-05

**Authors:** 


**Mathematic Law of Digestive Activity.** Pawlow has shown that, approximately, the amount of gastric juice secreted in a given time and the duration of digestion, vary as the square root of the quantity of food.

